# Age, morphology, and environmental variation shape movement behaviour syndromes in a riverine fish – golden perch (*Macquaria ambigua*)

**DOI:** 10.1186/s40462-026-00672-8

**Published:** 2026-06-12

**Authors:** Joshua Whiley, Luke Carpenter-Bundhoo, Doug Harding, Kate Hodges, Ryan Woods, Glenn B. McGregor, Jonathan C. Marshall, James Fawcett, Colin L. Burke, Kyne Krusic-Golub, Brien Roberts, Gavin L. Butler, Mark J. Kennard

**Affiliations:** 1 Australian Rivers Institute, Griffith University, Brisbane, QLD 4111 Australia; 2 Department of Local Government, Water and Volunteers, Brisbane, Qld Australia; 3 Department of the Environment, Tourism, Science and Innovation, Brisbane, Qld Australia; 4 Grafton Fisheries Centre, Department of Primary Industries and Regional Development, Grafton, NSW Australia; 5 Fish Ageing Services Pty Ltd, PO Box 396, Portarlington, VIC 3223 Australia; 6 Fisheries Research, Department of Agriculture and Fisheries, Northern Territory Government, Darwin, NT Australia; 7 Research Institute for the Environment and Livelihoods, Charles Darwin University, Darwin, NT Australia

## Abstract

Understanding the scale and characteristics of animal movements allows for effective species management. These movement characteristics can vary widely within a species, so there is a need to understand and account for the spectrum of movement behaviours within a population. Movement ecology studies of freshwater fish predominantly aim to uncover environmental correlates of population-wide movement behaviours. However, few studies consider the specific movement types that may exist within a species or population. This is somewhat surprising considering the importance of fish movement and migration in determining population characteristics and species persistence over time. In this study, we used acoustic telemetry to investigate the role and extent of intrapopulation variation in the movement behaviour of golden perch (*Macquaria ambigua*, Percichthyidae) in the Condamine-Balonne River system, Australia. After tracking 132 individuals between 2018 and 2025, we employed a novel approach to characterise annual fish movement behaviour into distinct movement groups using a suite of movement data metrics. These groups were then compared to morphological and environmental variations, assessing group membership. We found strong variation in the movement behaviour of golden perch and identified five distinct movement groups. The proportion of individuals in each movement group varied with hydrological alteration and demonstrated behavioural plasticity within the population, possibly in response to the increased density within weir pool habitats. Fish that exhibited strong home-site residency and low mobility (under 5 km linear range) were most prevalent, and only a small number of fish (*n* = 18) moved more than 50 km per year. Higher mobility was associated with larger body size (length and weight) and increased age. Future management actions for freshwater fish may benefit from recognising the diverse movement syndromes present within a population. Integrating these behavioural groups, and their differing requirements, into environmental flow management could better preserve key ecological functions and strengthen population resilience.

**Supplementary Information:**

The online version contains supplementary material available at 10.1186/s40462-026-00672-8.

## Introduction

Animal movement is a major driver of ecosystem functioning and composition, and forms a fundamental pillar of ecological research [1]. Quantifying how and why animals move within a heterogeneous environment allows researchers to better understand factors such as habitat use, animal behaviour and migration patterns [2, 3]. Intraspecific variation in movement behaviour is ubiquitous in nature. For example, many species exhibit partial migration (also known as facultative migration), where populations comprise sympatric migratory and resident individuals [4, 5]. Individuals may also differ in the timing, distance, direction, and duration of movements [6]. Similar movement characteristics that are shared by groups of individuals are known widely as ‘movement syndromes’ [7–9]. Such phenotypic diversity has profound consequences for individuals, populations and ecosystems [6, 10]. The environmental factors and individual traits that shape life history expression must therefore be well understood for targeted population management and conservation efforts.

In spatially constrained systems, such as rivers, an understanding of species’ resource requirements and how they access those resources is of particular importance. Fish movements may involve small general home range movements and foraging, to large-scale dispersal and migratory movements spanning hundreds to thousands of kilometres [11]. These movement behaviours can vary widely between individuals and populations within a species [12, 13]. River fragmentation (e.g. due to dams and weirs) and flow alterations (e.g. due to water extraction and interception) may also affect fish movements by causing barriers to longitudinal connectivity and changing behavioural cues stimulating movements [14–16]. Understanding how movement characteristics vary within and between populations, and which factors influence these variations, is important in ensuring that management practices, such as environmental flow provisions or harvest limits, effectively enable critical life history movements to occur.

Under current management practices, whole species or population behaviours are typically considered, but little attention is given to the more complicated possibility of broad behavioural groupings within species. The restricted movement paradigm [17] posits that populations of riverine fishes are often dominated by sedentary individuals, while a smaller proportion exhibit high mobility. This behavioural dichotomy has been widely reported across multiple taxa, including Percichthyids and Cyprinids [10, 18, 19]. The persistence of such behavioural variation within populations suggests that alternative movement strategies may confer context-dependent fitness advantages [20]. Individuals who move more often may encounter and capitalise on advantageous conditions, escape adverse conditions or density-dependent stressors such as competition, predation, or disease, and contribute to dispersal and recolonisation processes. Alternatively, individuals that remain in suitable habitat may not reach areas with potentially superior conditions, yet they may reduce exposure to adverse environments and, by staying in densely populated areas, enhance their reproductive opportunities [6]. While variation in movement strategies can increase population resilience in unpredictable environments [21], it can complicate management interventions, such as environmental flow releases, underscoring the need to understand both the extent and nature of this variation.

Movement behaviour is also shaped by life-history processes, with many species exhibiting pronounced ontogenetic shifts across developmental stages [22]. This often manifests in the transition between habitats during their life stages, such as the use of vegetated shallow areas by larval zander (*Sander lucioperca*) before movement to deeper, more complex habitats with maturation [23], or the initiation of spawning migrations upon reaching reproductive maturity, for example, as observed in European barbel (*Barbus barbus*) [24]. However, for facultatively migratory species, the extent to which individual traits (e.g., length, weight, and age) influence the expression of movement behaviour, and how this varies across environmental contexts, remains poorly understood [10].

Golden perch (*Macquaria ambigua*) is a relatively long-lived (common to 20 years, mature at 3–4 years), facultatively potamodromous species in the family Percichthyidae, whose movement ecology has been the focus of multiple studies over the last 60 years [25–28]. It is generally considered to be a highly mobile species, and has been shown to undertake large-scale movements over hundreds of kilometres in a single event [26, 27], and in rare cases over 1000 km (cumulative distance) [26, 27, 29]. Such movements are generally associated with upstream dispersal of juvenile fish following the downstream drift of eggs and larvae, and spawning migrations, which typically occur in spring or summer following an increase in flow and river level [30, 31]. In contrast to this, some studies have shown strong site fidelity and extremely limited movement in golden perch, with 80–100% of tagged fish moving less than 5 km, and many under 0.5 km [32, 33]. Although large-scale movements have been frequently reported, it is important to note that such movements were not undertaken by the entire population. The apparent behavioural dichotomy observed across studies examining movement and migration in this species suggests that their behaviour may be broadly aligned with the restricted movement paradigm [17, 19]. However, the environmental and intrinsic factors that influence whether an individual will express predominantly ‘sedentary’ or ‘mobile’ behavioural types, and whether more nuanced delineations exist, remains unclear.

Despite numerous studies examining golden perch movement behaviours across their large distribution [34, 35], intra-population variation in movement types and the influencing factors that may drive this behavioural diversity are not well understood. Marshall et al. [15] analysed golden perch movements and classified them into mobile and sedentary movement groups. However, further delineation of possible movement groups or the factors influencing group membership is not yet well understood.

In this study, we use golden perch as a model species to investigate intra-population variation in movement behaviour and the traits that influence its expression. We aim to characterise intra-population variation in the movement behaviour into distinct syndromes based on their movement characteristics. We then examined how biological and environmental factors related to movement syndrome composition. We used acoustic telemetry to track 132 individuals over 7 years throughout the Condamine-Balonne River, a highly variable and regulated dryland river in inland Australia.

## Methods

### Study area

**Fig. 1:**
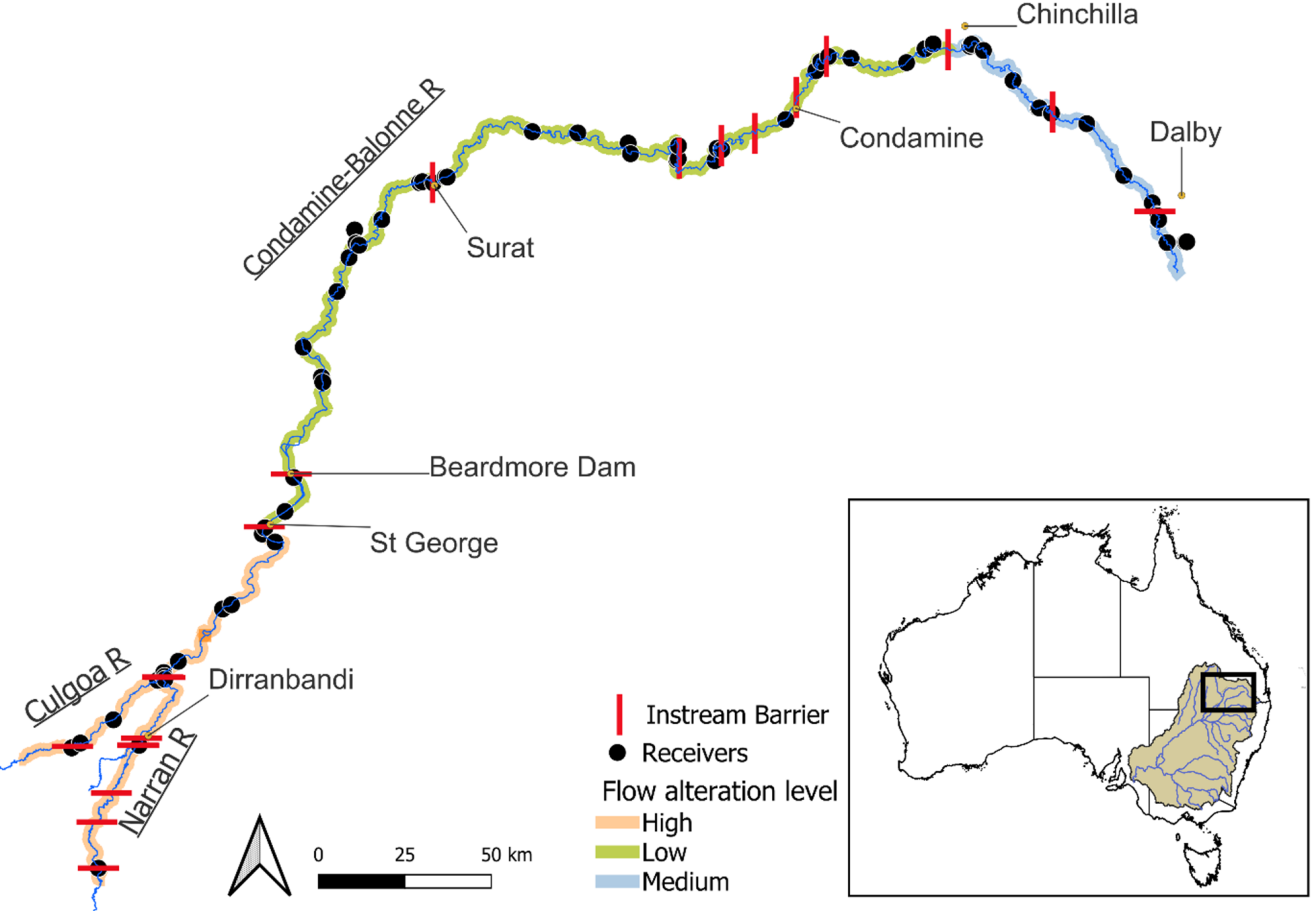
Acoustic telemetry array in the Condamine-Balonne River system, located in the northern Murray-Darling Basin, Australia (inset map). Black circles indicate the positions of acoustic receivers and red lines represent potential barriers to fish movement due to dams and weirs (identified by [37]. The spatial extent of flow alteration as determined by Harding et al. [36] in the Condamine-Balonne River is indicated with colours (three levels: low, medium, high)

The study area covers ~ 870 km of the Condamine-Balonne River in the northern Murray-Darling Basin in inland Australia. The Condamine-Balonne River is a dryland river with highly variable flow regimes and intense drought and flood periods. It has a catchment area of 143 900 km^2^ and a mean discharge of 2904 ML/day (2020–2025) at St. George (Fig. 1). The Condamine-Balonne River has experienced substantial flow alteration since the 1960s due to dam and weir development for water storage and extraction for irrigation. The study area was categorised into three levels of river flow alteration based on a reference comparison: low, medium, and high (Fig. 1). The study reach was categorised into three levels of river flow alteration (low, medium and high; Fig. 1) based on hydrological analyses presented in Harding et al. [36]. This analysis quantified differences in key ecologically relevant flow metrics calculated from simulated daily discharge time series (1900–2022) for pre-development and full water resource development conditions. The full-development scenario represented the total maximum entitled water extractions and dam operations, whereas the pre-development scenario assumed no extraction or infrastructure within the catchment (see [36]; for more details). The low alteration area stretches from Chinchilla Weir to St George (~ 480 km river length) (Fig. 1) and represents the smallest differences in flow metrics between pre-development and full-development conditions. The medium alteration area, extending from Loudouns Weir in Dalby to Chinchilla Weir (~ 160 km river length), is characterised by an increased frequency and duration of low flow periods and exhibits changes to the natural flow regime at a smaller scale than the high alteration area. The high alteration area was downstream of Jack Taylor Weir, extending downstream from St George to the most downstream receivers in the Narran and Culgoa Rivers (~ 230 km of tracked river length). The magnitude, frequency, and duration of flows within this area are heavily reduced compared to pre-development conditions, with mean daily flows lower and subject to greater annual variability. Throughout the approximately 870 km river stretch, there are 19 barriers (dams and weirs), ranging from 0.6 to 12.2 m wall height (Fig. 1) [37]. The reach length between barriers ranged from 0.4 to 159.1 km (mean + SD = 49.5 *±* 39.9 km). Only three of the weirs in the study reach have fish passage devices (fishways). Except for Beardmore Dam, all these barriers periodically drown out during elevated flow events (water level downstream of barrier rose to a height where it is equal to the level upstream) and are assumed to be passable to fish, between 0 and 18.5% (x̄ across all barriers = ~ 10%) of the study period (April 2018 - May 2025).

### Fish collection, acoustic tagging and telemetry

Between April 2018 and May 2025, 371 golden perch were captured, tagged, and released at 18 sites across the Condamine-Balonne. Fish collection was undertaken with a boat-mounted electrofisher using standardised settings. Voltage, frequency, and duty cycle were adjusted at each site depending on the local environmental conditions to ensure minimal effects on fish and other biota. Tagged fish were measured (standard length and total length ± 1 mm) and weighed (± 1 g). Transmitters (Innovasea V13–1X and V9–2X) were surgically implanted in sub-adult and adult fish, classified by body size, using methods adapted from Butler et al. [38] and described in Harding et al. [36]. Fish movement was monitored by an array of 57 acoustic receivers (Vemco VR2W; Innovasea Halifax) placed at regular intervals (min = 0.2 km, mean + SD = 14.2 *±* 14.3 km) throughout the study reach (Fig. 1). The mean distance between receivers was 13.5 *±* 12.9 km in the low alteration area, 11.8 *±* 10.0 km in the medium alteration area and 17.2 *±* 19.0 km in the high alteration area. See Harding et al. [36] for further details on sampling and acoustic tracking methodology. The receiver array recorded a binary presence/absence for each tagged fish detected within the maximum reception range (~ 300 m) of a given receiver.

Of the 371 tagged golden perch, 345 were detected in the array after release. Fish that were detected on only one receiver and displayed no detectable movements were assumed to be alive and sedentary and were retained in the analyses, provided that detections were discontinuous, thereby indicating the individual was locally active. To ensure that classification of movement behaviour was based on sufficient data, and to allow for accurate calculation of average yearly movement characteristics, fish with fewer than 20 detections, or those that were detected in the array for less than nine months (~ 10% of the study period), were removed from the analyses (*n* = 239 of 371 tagged golden perch removed). The remaining 132 golden perch were retained for movement behaviour analysis (Table 1, Supplementary Table 1). Fish that were not detected after release and those with limited detections over the study period may be a result of tagged fish leaving the array, the expulsion of tags, mortality, or due to sedentary behaviours in reaches beyond the reception range of the receivers.

**Table 1:** Details of tagged golden perch from three areas of varying river alteration in the Condamine-Balonne River

River alteration level	*n*	Mean standard length (mm) (range)	Mean weight (g) (range)
Low	81	315 (245–400)	811 (342–1938)
Medium	22	336 (225–412)	990 (318–1538)
High	29	277 (185–410)	544 (140–1446)

### Data analyses

#### Fish movement metrics

The river path and location of each acoustic receiver were digitised in *QGIS* (ver. 3.26, see www.qgis.org). This spatial object was then converted into a transition layer (connectivity matrix defining movement between adjacent river segments), and within this layer, the distances of the shortest river path between subsequent relocations of individuals were generated using the *gdistance* package [39] in R (ver. 4.5.1, R Development Core Team; http://www.r-project.org). The distance between fish detections was then calculated, and the daily sum of total hydrographic distance moved was calculated for each fish. Detections in the first 24 h post-release were removed from further analysis to account for any potential abnormal movements, as a result of the capture and tagging process [14]. The summed daily detection data were then used to calculate 16 movement metrics for each of the detected individuals. These captured displacement (cumulative distance [km], cumulative upstream distance [km], cumulative downstream distance [km], linear range [km], and proportion of accessible river used [%]); movement intensity (movement frequency [n], rate of movement [mean and max, km h⁻¹], continuous distance moved [median and max, km], and continuous duration of movement [median and max, days]); and motivation to move (proportion of movements across four hydrological limbs: base, rising, peak, and receding [%]; Table 2, Supplementary Fig. 2). The latter movement metric was calculated using hydrological data sourced from the Department of Local Government, Water and Volunteers [40] and WaterNSW gauging stations [41] from ten locations (Supplementary Table 2). The data included the daily flow discharge (megalitres per day, ML/day) from 16/03/2012 (the earliest available date across all gauges) to 07/07/2025. The *hydrostats* package [42] in R was then used to calculate each flow limb. The mean baseflow magnitude for each of the water monitoring gauges was calculated using the Lynne-Hollick filter [43]; any day where daily discharge did not exceed the calculated base flow was categorised as ‘base’. For each gauge, any day that experienced an increase in flow from the previous day and exceeded the base flow value was categorised as a ‘rising’ flow, and any day that experienced a decrease in flow from the previous day without falling below the calculated base flow was categorised as ‘receding’. ‘Peak’ was used to describe days when a rising flow had reached and sustained its maximum discharge before receding. To account for differences in monitoring duration, each movement metric was standardised by the number of active years in the array, resulting in an average per-year value.

**Table 2:** Definitions and explanatory values for the 16-movement metrics used to classify movement behaviours of golden perch in the Condamine-Balonne River

Movement metric	Value range (Mean ± SD)*	Definition	Abbreviation
n. detected movements	0–25.92 (2.83 ± 4.08)	Number of times the individual was detected on a different receiver than their previous detection (n)	nodm
Cumulative distance	0–197.18 (23.8 ± 38.29)	Total distance moved (km)	cum_dist
Upstream cumulative distance	0–167.37 (11.54 ± 23.46)	Total distance moved travelling upstream (km)	upstream
Downstream cumulative distance	0–153.19 (12.26 ± 22.47)	Total distance moved travelling downstream (km)	downstream
Linear range	0–85.3 (10.76 ± 18.91)	The distance between the most upstream and downstream detection (km)	range
Rate of movement		Distance of movement divided by time taken to complete movement (km/hr)	rom
Mean	0–1.6 (0.23 ± 0.29)		
Max	0–3.55 (0.51 ± 0.68)		
Continuous distance moved		Distance moved in a continuous movement. Movement streaks are ended when an individual is detected on the same receiver on consecutive days, or remains undetected for > 3 days (km)	cont_dist
Median	0–70.53 (8.83 ± 13.83)		
Max	0–167.37 (13.74 ± 23.85)		
Continuous duration moved		Duration of a continuous movement. Movement streaks are ended when an individual is detected on the same receiver on consecutive days or remains undetected for > 3 days (days).	cont_dur
Median	0–5.39 (1.91 ± 1.63)		
Max	0–11.01 (2.61 ± 2.4)		
Percentage of accessible river used	0–336.89 (24.86 ± 51.62)	Linear range divided by the accessible river extent (distance between nearest upstream and downstream barrier [37] from the receiver on which an individual was most commonly detected; %).	area_used
Proportion of movements by hydrological ‘limb’		Flow from each day was categorised into one of four (base, rising, peak, and receding) hydrological limbs, and the proportion of each individual’s movements by limb was calculated (%).	
Base	0–100 (28.42 ± 31.7)		mov_base
Rising	0–100 (11.93 ± 20.56)		mov_rise
Peak	0–33.33 (2.25 ± 6.67)		mov_peak
Receding	0–100 (13 ± 18.62)		mov_recede

*Movement metric values were standardised by each individual’s time in the array and converted to the average per year

#### Classification of fish movement behaviours

A hierarchical cluster analysis of individuals was performed using the 16 movement metrics to identify groups of golden perch individuals with similar movement behaviours, calculated using the *vegan* package in R [44]. The cluster analysis was performed using Ward’s Linkage criterion based on a Euclidean distance matrix, and the optimal number of clusters was determined using the *factoextra* package and the elbow method [45, 46]. The effects of tagging time and location on movement behaviour classification were visually assessed using abacus plots and river position-through-time plots, separated by movement syndrome.

#### Fish biological attributes potentially explaining fish movement behaviours

Several biological attributes were evaluated for the potential to explain differences in fish movement behaviours defined by the cluster analysis. These included individual fish standard length (mm), weight (g), age (years), and body condition.

Fish age was attributed to fish based on length at the time of tagging using an age-length key. This was developed as described in Isermann and Knight [47] using the *FSA* package in R to predict the age of fish based on their lengths [48] (Supplementary Fig. 3). See supplementary Table 1 for the estimated ages of all golden perch used in this study.

The body condition of tagged golden perch was calculated using a standardised residual condition index, as described in Pope and Kruse [49]. Length and weight data from 586 golden perch captured in the Queensland Murray-Darling basin [50] were log-transformed, and their linear regression was calculated. The expected weight of each tagged fish in this study was then calculated using the linear regression formula, and the residual of the difference between the observed and expected weight for each fish was calculated.

#### Environmental attributes potentially explaining fish movement behaviours

Several environmental variables describing anthropogenic flow alteration and river fragmentation caused by weirs were quantified to evaluate their potential influence on observed differences in fish movement behaviours. The duration of time each of the 19 identified barriers was passable was estimated using hydrological data in conjunction with minimum barrier drown-out data [37]. In addition, the proportion of the accessible river extent each individual moved through was calculated (i.e. distance between the nearest upstream and downstream barrier from the receiver on which each fish was most commonly detected; Table 2).

#### Relating fish movement behaviours to biological and environmental attributes

Variation in age, standard length, body condition, and weight between the generated movement behaviour syndromes was initially investigated visually. Between-group differences in weight, length, age, and condition were assessed using Wilcoxon signed-rank tests and Kruskal-Wallis tests. Where a significant result was returned by these tests, Dunn’s post hoc tests with Bonferroni-adjusted *p-*values were used to identify specific inter-group differences. Differences in the proportions of individual movement syndromes among river alteration areas were assessed separately using Chi-square tests.

## Results

### Movement syndromes

**Fig. 2:**
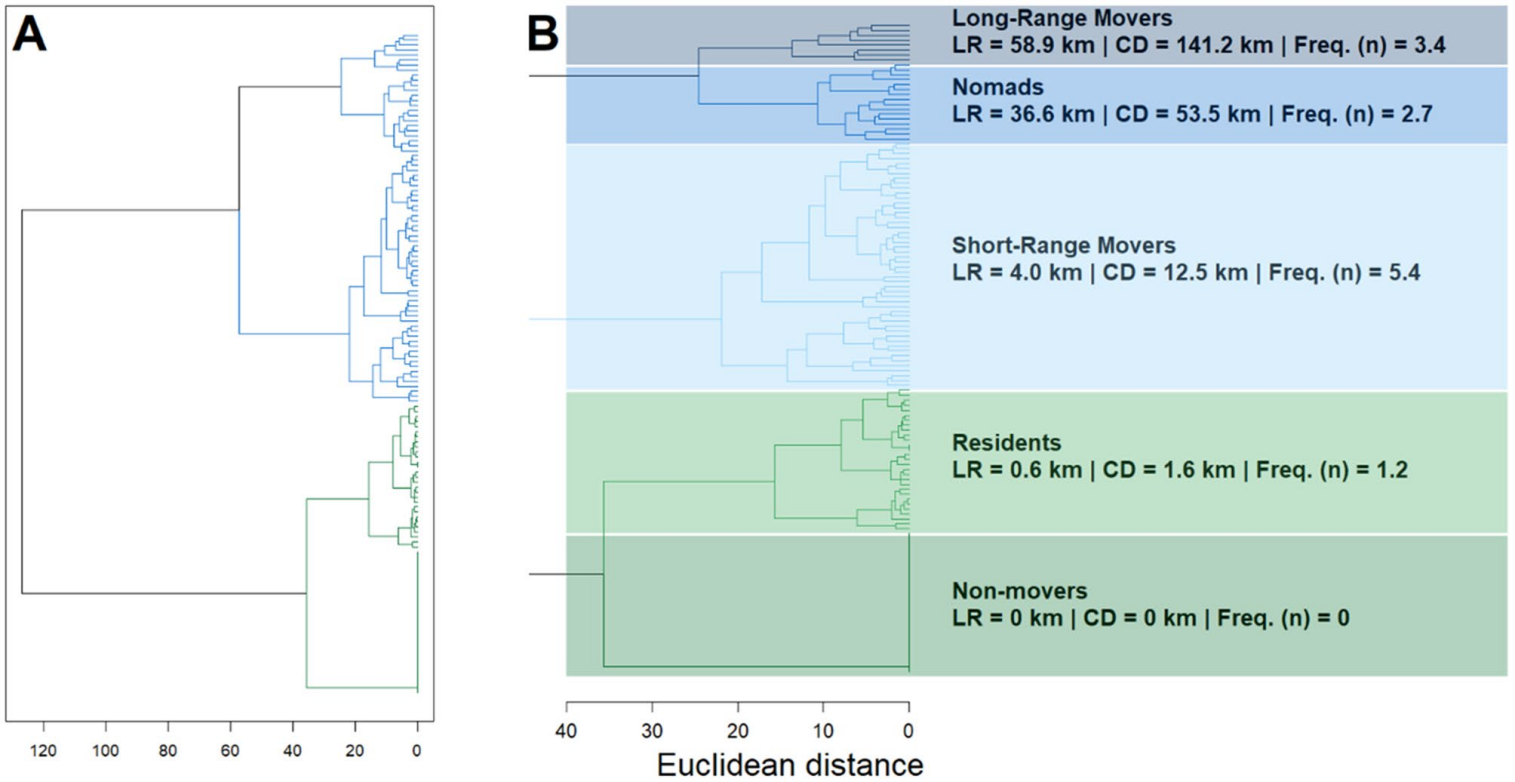
(**A**) Dendrogram of the two broad golden perch movement syndromes, and (**B**) five fine movement syndromes presented with the three most informative metrics. Sedentary *n* = 58 (Non-movers *n* = 26, Residents *n* = 26) and Mobile *n* = 74 (Short-range movers *n* = 50, Nomads *n* = 16, and Long-range movers *n* = 8). Median yearly linear range (LR; km), median yearly cumulative distance moved (CD; km), and median yearly movement frequency (*n*) for each of the movement syndromes are shown on the right. Branch colours denote movement syndromes

**Fig. 3:**
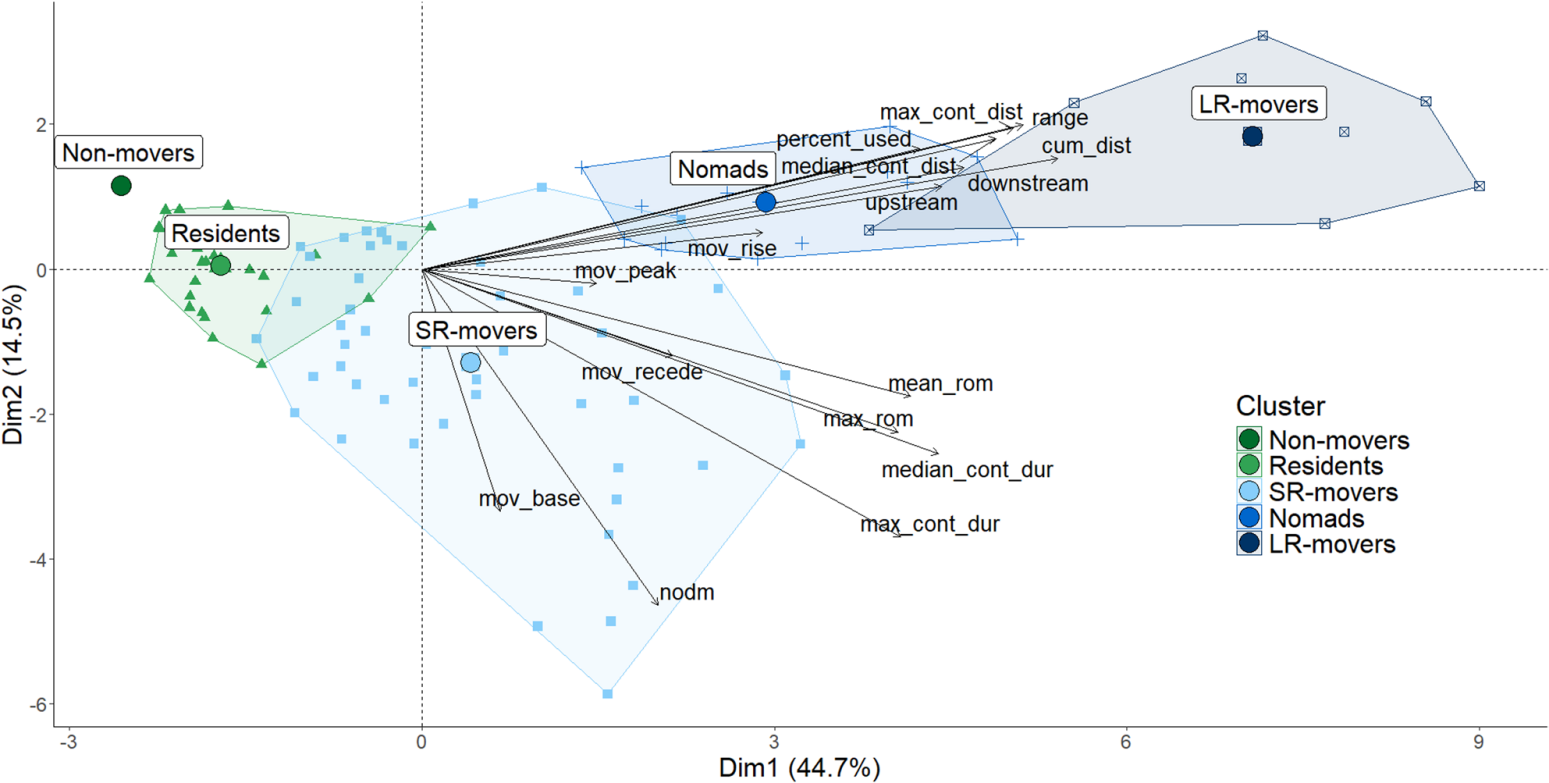
Principal components analysis (PCA) of golden perch movement metrics, with each of the 16-movement metrics overlayed. Arrows indicate the direction and strength of each variable’s contribution to the principal components. Movement syndromes are labelled and denoted by point colour and shape. Centroids are shown as the largest point in each ellipse

Examination of golden perch movement characteristics revealed two broad groups, which were then further split into five fine movement syndromes (Figs. 2 and 3). The broad groups were: *Sedentary*, which displayed either no detectable movements or only minimal movements, resulting in limited cumulative distances moved, small linear ranges, and infrequent movements; and *Mobile*, which comprised several life strategies that differed in the mode and extent to which they moved. The composition of the golden perch was relatively balanced between these two movement groups, with 74 fish classified as *mobile* and 58 as *sedentary* (Fig. 2).

**Fig. 4:**
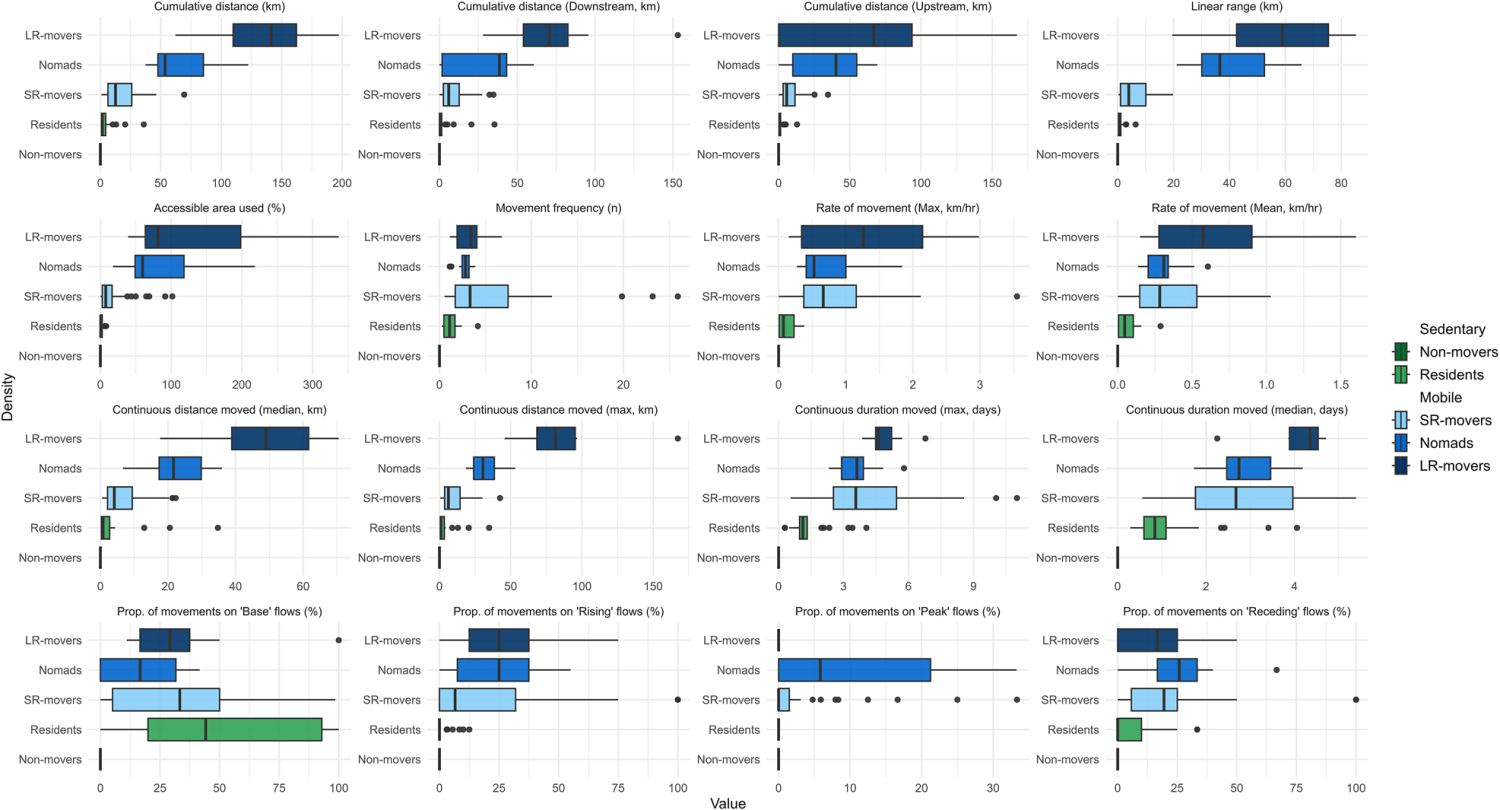
Boxplots showing variation in 16 movement metrics among the five fine-scale movement syndromes of golden perch from the Condamine-Balonne River. Each facet displays one of the movement metrics used to categorise golden perch movement behaviours (e.g., cumulative distance, linear range, rate of movement). Variables are standardised by time in the acoustic array and displayed as the average value per year. Boxplot colour denotes movement group, with fine-scale movement syndromes in blue belonging to the mobile movement group and those in green belonging to the sedentary movement group. The median is represented by the thick black line in each box plot. Outliers are shown as individual points

The fine movement syndromes that comprised the *sedentary* group were (1) *non-movers*, who displayed no detectable movements throughout the study, but remained active in the array for at least nine months; and (2) *residents*, who displayed strong site residency with little movement outside of this area, resulting in small linear ranges (LR; median = 0.6 km), low cumulative distances travelled (CD; median = 1.6 km), and infrequent movements (Figs. 2 and 4).

The *mobile* group had three distinct movement syndromes. (1) *short-range movers*, who showed strong site residency, similar to that of the *residents*, but who made frequent short-range movements across a larger home range area, with some examples of philopatric behaviour, resulting in a larger cumulative distance (median > 7**x** that of the residents; 12.5 km; Fig. 4) and linear range (median = 4.0 km). (2) *Nomads*, which were characterised by a lack of site residency, with irregular movements across an intermediate range that utilised a higher proportion of the barrier-free river available to them, resulting in a substantially higher cumulative distance and linear range (median CD = 53.5 km, median LR = 36.6 km; Fig. 4). (3) *Long-range movers*, characterised by large range movements throughout the river system (max 197 km/year), which covered an extended linear range (median LR = 58.9 km; Fig. 4).

**Fig. 5:**
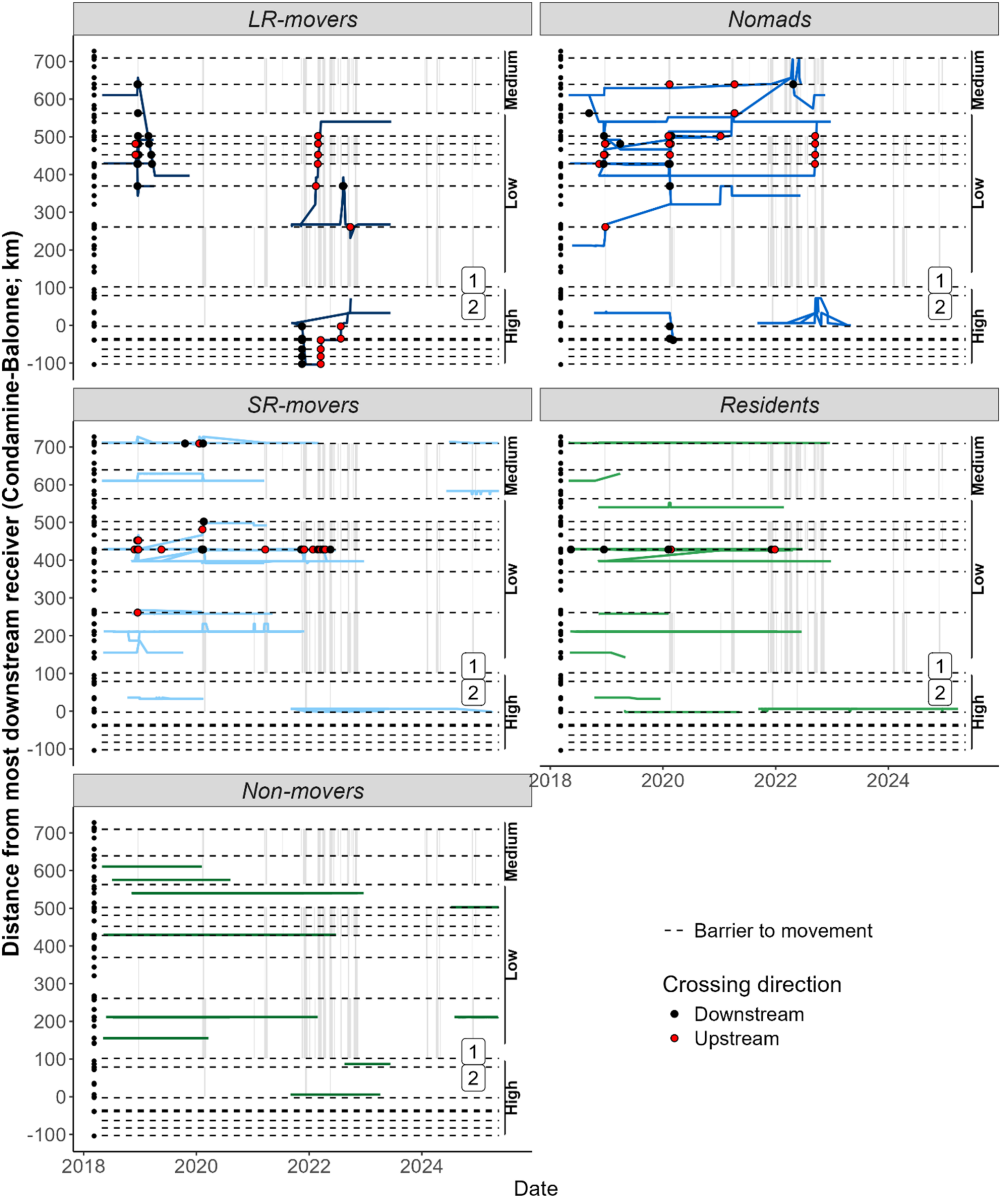
River position of tagged golden perch in the Condamine-Balonne River through time, separated by five identified movement syndromes. The value of 0 on the y-axis represents the most downstream receiver in the Condamine-Balonne River, and negative values align with barrier/receiver location in the Narran River (see Fig. 1). Receiver locations are indicated by black points to the right of the y-axis. Dotted horizontal lines show the position of barriers throughout the river system, and facing y-axis labels “low”, “medium”, and “high” indicate the extent of each river alteration zone. Grey vertical bars show periods where barriers are drowned out and assumed to be passable to fish. Points indicate barrier crossing events (red denotes upstream crossings and black denotes downstream crossings). Numbers 1 and 2 indicate barriers that fish did not cross during the study period: (1) Beardmore Dam, (2) Jack Taylor Weir. Barriers (*n* = 2), receivers (*n* = 5) and individuals (*n* = 3) in the Culgoa River were excluded from the figure

The *short-range movers* syndrome was dominant (37.8%). *Non-movers* and *residents* were the second most prevalent movement syndromes (equally at 21.9%). The proportion of highly mobile golden perch was low, with only 16 (12.1%) fish being classified as *nomads* and 8 (6%) as *long-range movers* (Fig. 4). All movement syndromes were observed across the majority of the study period and were spatially dispersed throughout most of the study area (Fig. 5; Supplementary Fig. 4). Barrier crossings were uncommon (*n* = 128 fish movement instances); however, drown-out events typically resulted in at least one fish crossing (Fig. 5). Upstream crossings were demonstrated at 17 of the 19 identified barriers by 29 fish, ranging in standard length from 245 to 380 mm. *Short-range movers* crossed barriers most frequently (*n* = 44), followed by *long-range movers*, *nomads*, and *residents*, with 38, 35, and 11 crossings, respectively (Fig. 5).

### Variation of morphological variables between movement syndromes

**Fig. 6:**
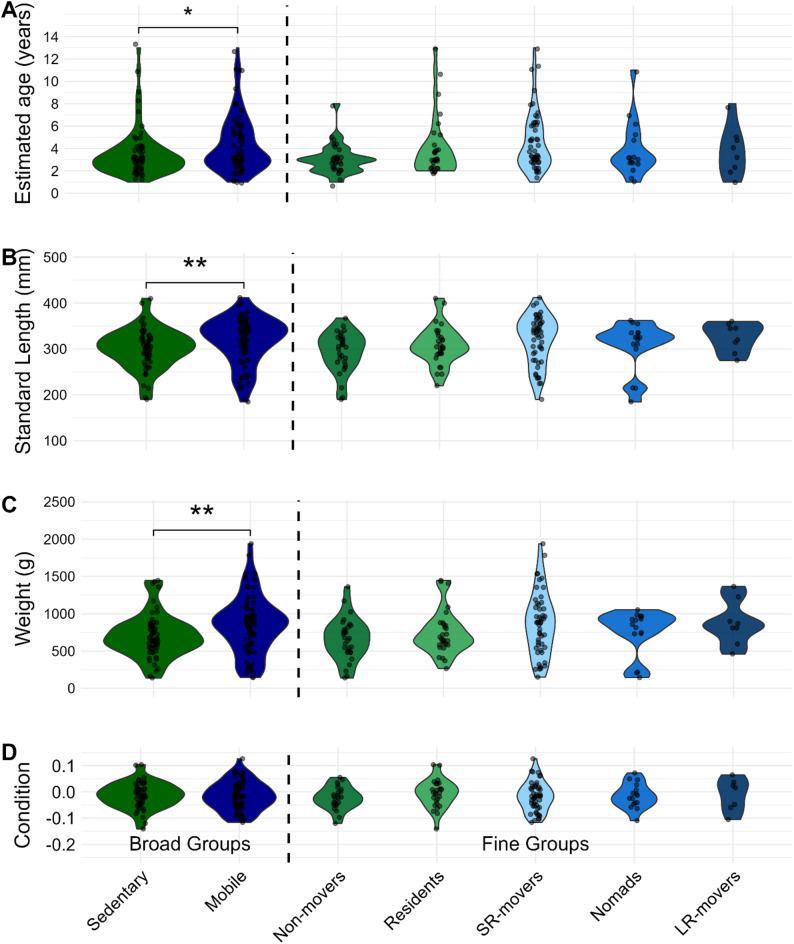
Distribution of golden perch morphological variables across two broad-scale and five fine-scale movement syndromes in the Condamine-Balonne River; (**A**) estimated age (years), (**B**) standard length (mm), (**C**) weight (g), (**D**) body condition. Violins left of the dashed vertical line represent the two broad movement groups (Sedentary and Mobile), while violins on the right of the dashed line represent the five fine movement syndromes (Non-movers, Residents, Short-range movers, Nomads, and Long-range movers). Points represent individual fish, and area colour denotes movement syndrome membership. Asterisks represent statistically significant differences between the compared syndromes (indicated by horizontal bars; *p* < 0.05 = *, *p* < 0.01 = **) based on Wilcoxon rank-sum tests (Broad movement groups) and Kruskal-Wallis tests (Fine movement syndromes)

The estimated age of all tagged golden perch ranged from 1 to 13 years, with a mean age of 4.0 ± 2.4 years (x̄ ± SD). *Mobile* fish were slightly older on average, with a mean age of 4.4 ± 2.54 years, compared to *sedentary* fish with a mean age of 3.6 ± 2.23 years (Wilcoxon; *p* = 0.03). Estimated age did not differ significantly between the five movement syndromes (*p* > 0.05; Fig. 6A). *Mobile* golden perch, on average, were significantly heavier (Wilcoxon; *p* < 0.01) and longer (Wilcoxon; *p* < 0.01) than *sedentary* fish, with a median weight of 879 g and a mean standard length of 329 mm, while *sedentary* fish had a median weight and standard length of 666 g and 304 mm. However, there was no significant difference in length and weight among the five movement syndromes (Kruskal-Wallis; *p* > 0.05; Fig. 6B and C). The condition index for golden perch ranged from − 0.39 to 0.13, with the mean body condition score being − 0.02 +/- 0.06 (x̄ ± SD). Body condition was similar between the broad-scale movement groups, with the *Mobile* group at -0.017 and the sedentary group at -0.014 (Fig. 6D). The average body condition was also fairly consistent across the five fine movement syndromes and showed no significant difference between the syndromes (Fig. 6D).

### Movement group membership by level of river alteration

**Fig. 7:**
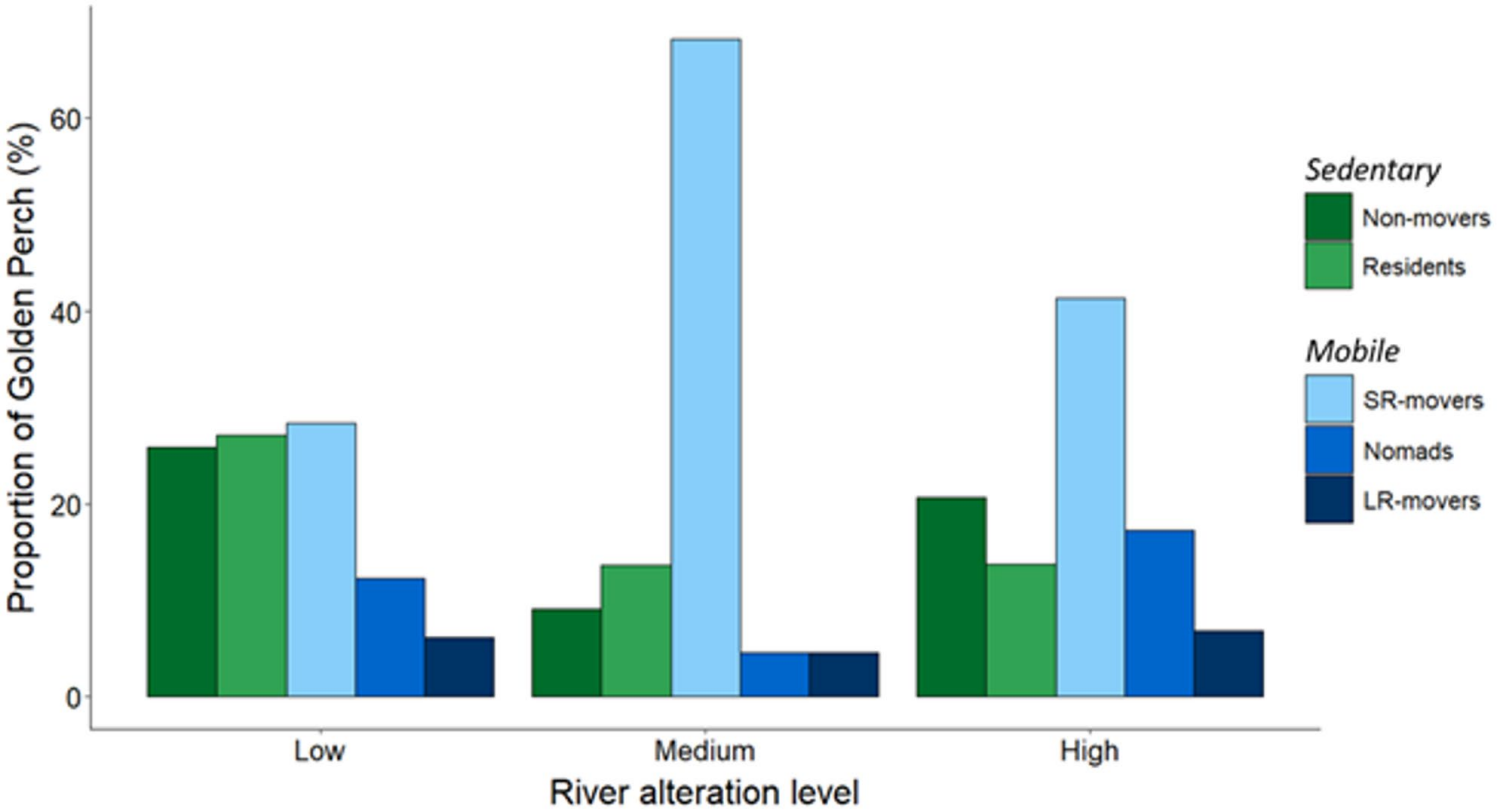
Composition of five golden perch movement syndromes (%) across three levels of river alteration in the Condamine-Balonne River. The movement behaviour group is denoted by bar colour (greens = sedentary, blues = mobile)

The composition of movement syndromes across all three areas of river alteration was dominated by the *short-range* movement syndrome, with the medium alteration area having the highest proportion of *short-range movers* (68%), followed by the high and low alteration areas. The proportion of *non-movers* was relatively consistent between the river alteration areas; however, there was a particularly low number of *non-movers* in the medium alteration area (*n* = 2). *Nomads* accounted for the fourth most prevalent behaviour group. This group was most prevalent in the high-alteration area, followed by the low and medium areas at 17, 12, and 5 per cent, respectively. *Long-range movers* accounted for the lowest proportion of fish, with 6% (*n* = 2) of the high alteration area, followed by the low alteration area at 6% (*n* = 5) and only 4% (*n* = 1) of the medium alteration area (Fig. 7). The proportion of *short-range movers* varied significantly with the level of river regulation (χ^2^, *p* > 0.003), with no significant statistical association between river alteration level and the other movement syndromes.

## Discussion

Understanding intra-population diversity in movement strategies is an emerging area of interest for the study and management of freshwater fauna, particularly in regulated and hydrologically variable river systems. Golden perch in the Condamine-Balonne River system displayed five distinct movement syndromes that illustrates the behavioural diversity that can be present within a single population. These distinct movement syndromes were characterised by highly varied movement behaviour strategies, encompassing sedentary individuals with strong home site residency and several mobile life history strategies. The information generated in this study could be used to inform a review of management practices for this important species to accommodate the diversity of movement syndromes.

In terrestrial and marine systems, further movement groups outside of ‘movers’ and ‘non-movers’ have been established for differing species, populations or ontogenetic stages [9, 51], but discretely defined movement classes are less prevalent in studies of freshwater organisms (e.g. [12, 52]). Traditionally, movement ecology studies investigating behavioural variation have assigned movement syndromes at the species level or quantified the proportion of movements of a given movement type [53, 54]. More recently, there has been a shift toward assigning movement syndromes at the individual level within species or populations [7, 55]. However, this approach has seen limited application in freshwater systems. This study contributes to addressing this gap by identifying and classifying individual movement syndromes in golden perch within a regulated river system, providing novel insights into behavioural variation and its potential drivers in freshwater fish populations.

Previous studies that aim to interpret variation in movement behaviours within populations of freshwater organisms have been limited to a few general movement metrics (cumulative distance, linear range, and movement frequency) when delineating movement syndromes [12, 52]. As a result, such studies may have a reduced capacity to detect fine-scale or multi-dimensional movement strategies. Our inclusion of additional metrics that quantify movement intensity and motivation may improve the characterisation and ecological understanding of movement syndromes. For example, *short-range movers* moved most frequently under base flows, likely reflecting routine foraging movements, while *nomads* moved most frequently on rising flows, likely utilising improved connectivity to access new habitat. This study advances knowledge of golden perch movement behaviour and population-level movement variation in freshwater organisms and underscores the ongoing need for more integrative research to fully characterise this complexity.

### Movement syndrome composition

In this study, tagged golden perch in the Condamine-Balonne River were mainly sedentary or undertook only relatively small movements, with strong home site residency. Specifically, 64% had an average yearly linear range of less than 5 km, and very few fish (*n* = 18) moved more than 50 km per year. The golden perch in this study did not demonstrate extremely large-scale movements, as reported in some previous studies encompassing part of our study area [29] and other parts of the Murray-Darling Basin that are also fragmented by dams and weirs [26, 27]. The distances moved by the few large-scale movers did, however, exceed distances reported in previous studies in the southern Murray-Darling Basin [33, 34]. The prevalence of *sedentary* and *short-range movers* is consistent with the ‘restricted movement paradigm’ and supports previous studies that demonstrated strong home-site residency and limited movement in riverine golden perch [32, 33]. The proportion of these less mobile individuals is likely even greater than presented here, as acoustic telemetry may be biased towards higher detections of mobile individuals [56, 57]. Our study revealed that 239 individuals (64% of all tagged fish) did not meet our criteria for inclusion in the movement syndrome analyses (based on minimum time in the array and detection frequency thresholds), though 89% of the removed fish (*n* = 213) were detected in the array at least once after release. Golden perch have been demonstrated to have high tag retention rates and low mortality rates from intraperitoneal tagging (overall success rate of 81% after 315 days; [58]), and no fish were identified as leaving the array via a terminal receiver. It is therefore most likely that most of the fish removed from the analyses were alive and occupying areas beyond the ~ 300 m maximum reception range of the receivers, which were spaced 14.2 km apart on average.

Although many individuals exhibited strong site residency and limited ranges, a considerable portion displayed more mobile behaviours. This was particularly evident in the *nomad* group, which made infrequent movements across an intermediate range, possibly in search of suitable habitat. The proportion of mobile golden perch in this study (56%) and in those of Marshall et al. [15] (62.4%) was also substantially higher than the global median proportion of mobile individuals reported for riverine fish species (median = 33%; [59]). The coexistence of sedentary and mobile strategies within this population mirrors patterns observed in many riverine fishes globally, including other percichthyids, cyprinids, and salmonids, where partial migration or mixed movement behaviours have been hypothesized to contribute to population resilience [36, 60, 61]. However, rather than facultative potamodromous migrations or variable diadromy, golden perch in this study are displaying a spectrum of movement syndromes that allow the population to access a broader range of environmental conditions in a highly variable environment.

### Variation in morphological traits among movement syndromes

Age, length, and weight predicted golden perch movement behaviours, with increased fish size and increasing age associated with more mobile movement strategies. The association of larger body size and greater mobility in golden perch aligns with ecological patterns observed in other freshwater taxa globally, where movement capacity is generally theorised to scale with energetic reserves and morphology [59, 62, 63]. Similarly, Minns, [64] documented similar size-related differences in mobility in 18 fish species across 25 rivers. The results from this study are supported by those reported by Barrow et al. [65] and Barrow et al. [66], which found that mobile fish were larger than their sedentary counterparts and suggested that increased lifetime movement may be linked to increased growth during early life in golden perch. However, as age is estimated from length in the current study, we are unable to determine how length at age varies between *movers* and *non-movers*. It is unclear whether the relationship between increased size and mobility observed here is a result of mobile movement strategies allowing movers to access favourable conditions that promote increased growth, or if obtaining a larger size reduces the risks associated with adopting more mobile movement strategies. Another potential explanation is that larger, better-conditioned individuals have a greater capacity to move and can more easily provide the energy necessary for reproduction, which is linked to migration in golden perch [31, 67]. Being able to estimate the age of tagged fish through a means that is independent of length, such as through otolith chronology or DNA methylation, would provide a better understanding of the relationship between growth, size, and movement behaviours.

Larger golden perch, which are more likely to be reproductively mature, are expected to display higher mobility as their reproduction is strongly linked with migration [30]. Spawning migrations in golden perch typically occur throughout spring and summer in response to increased flow and overbank flows [31]. In this population, movement was generally associated with increased summer flow events [36], which resulted in barrier drown-out. Although mobility increased with body size, there was no clear ontogenetic shift associated with maturity. The mean estimated age of both the Mobile (4.4 ± 2.54 years) and Sedentary (3.6 ± 2.23 years) groups was close to the age at maturity for golden perch (males: 3 years; females: 4 years [68]). It is also possible that the increase in mobility observed in this population in response to summer flows [36] is not related to spawning migration, but rather that these periods of increased flow promote greater connectivity, and such flow events rarely occur in winter months during this study.

The presence of intra-population variation in movement behaviour in the Condamine-Balonne suggests that such benefits gained from adopting a mobile life strategy, or remaining sedentary, are not critically important for individual success and both may be viable. However, the high hydrologic variability of this system provides a variety of habitats that promote the adoption of varied life-behaviour strategies that may promote population persistence [21, 36]. The presence of sedentary individuals, that likely experience lower individual risk, may represent a stable core of the population capable of enduring prolonged periods of unfavourable conditions, such as drought [6, 15]. Conversely, the smaller proportion of highly mobile individuals may also support population persistence by utilizing beneficial conditions, facilitating dispersal, and promoting gene flow [69]. Together, these complementary strategies may enhance population stability through an ecological portfolio effect, where the diversity of behaviours buffers the population against environmental variability [21, 70]. Despite the apparent viability of adopting either a mobile or sedentary life strategy at the individual level, and the potential population-wide benefits of having varied movement strategies, we are unable to estimate how this may differ from pre-development conditions or affect golden perch populations long-term.

It is important to consider that post year two, this study was conducted during a period of higher discharge. Such hydrological conditions generally promote greater system-wide productivity and habitat connectivity, while mitigating density-dependent stressors, which may disproportionately benefit one movement syndrome over another [16, 71]. For example, improved conditions throughout the system as a whole may remove some of the incentive to move in search of more favourable conditions; whilst conversely, during periods where conditions are less favourable, the incentive to move to more favourable areas or refuge areas provides a much larger potential benefit [6, 72]. The potential costs and benefits of sedentary and more mobile life strategies will likely result in a different composition of the movement syndromes within a population during periods of strongly contrasting flow regimes [15]. It is also possible that individuals may shift between movement syndromes in response to periods of different environmental conditions or across life stages [73]. The period that each of the individuals in this study is tagged only represents a portion of its life and inhibits our ability to understand potential intra-individual variation in movement behaviour. Other approaches such as otolith-derived lifetime movement histories can help elucidate, albeit at much larger spatial scales than the fine-scale information presented here. Further research into the effect of environmental conditions on the composition of movement syndromes, and of potential intra-individual movement syndrome plasticity, would allow for a better understanding of this species’ movement behaviour.

### Effect of river alteration and barriers on movement syndrome composition

The presence of in-stream barriers in many systems prevents golden perch from undertaking large movements [25]. In the Condamine-Balonne, the composition of movement syndromes does not appear to be driven primarily by the physical restriction of in-stream barriers to movement. Golden perch in this study were subject to a similar range of environmental variations and restrictions from instream barriers yet displayed a range of movement syndromes. Fish that exhibited sedentary movement behaviours generally used relatively little of the barrier-free river that was available to them (53% of fish used less than 5% of their available space). Due to the wet phase climate conditions during the study, opportunities to cross barriers occurred relatively frequently (~ 10% of the study period), but such opportunities rarely resulted in an individual moving past a barrier. Fish ranging from 245 to 380 mm undertook upstream barrier crossings during these periods, demonstrating that upstream passage is possible; however, high water velocity during drown-out periods may have inhibited the ability of smaller fish or those in poor condition to pass in an upstream direction. Conversely, individuals who exhibited high mobility behaviours used a much greater proportion of the barrier-free river available to them and also undertook several barrier crossings in both up and downstream directions. This suggests that the prevalence of sedentary behaviours within this population was likely a result of choice rather than an inability to move. However, individual movement behaviour may ultimately reflect the costs and benefits of moving versus remaining resident with respect to individual fitness. In an altered system like the Condamine-Balonne, the costs of moving may be higher, and the benefits lower, than would be the case in an unmodified system. Thus, even though the majority of individuals can theoretically pass barriers at various stages, the proportion exhibiting sedentary movement behaviours may still be unnaturally high.

Observations suggest that while physical restriction on movement imposed by instream barriers is not the primary driver of movement syndrome composition, such barriers may influence fish motivation to move. Individuals classified as *residents* and *short-range movers* were commonly located just upstream of in-stream barriers, likely utilising the somewhat stable conditions provided by weir pools. Fish in these areas had a demonstrated capacity to move, with several fish making a downstream crossing of the barrier and exiting the weir pool, only to undertake an upstream crossing and return during the same drown-out period. The concentration of less mobile individuals near weir pools reflects a pattern reported across many fragmented rivers globally, where lentic conditions upstream of weirs or dams provide relatively favourable conditions for lentic species [74, 75]. This pattern was particularly pronounced in the medium and high river alteration areas that contain a high density of weirs and associated pool habitats that are likely conducive to reduced mobility. In the low alteration area, where this habitat type is less prevalent, there was a more balanced composition of movement behaviours. The dominance of short-range movers in the high and medium alteration areas may be the result of behavioural adaptation to the altered hydrological conditions throughout this system. Such behavioural plasticity in golden perch at a population scale has not been reported previously in response to anthropogenic influences; however, golden perch have a generalist life history and behavioural modification due to environmental causes, such as drought, is well documented [34, 76]. Such behavioural plasticity in golden perch, potentially in response to habitat modification, can help managers to better understand the impacts of river alteration.

### Implications for management

Our study suggests that there is significant intra-population variation in the movement behaviour of golden perch in the Condamine-Balonne River. This behavioural flexibility provides both favourable implications and challenges for the future management of the species in highly altered river systems. Generalised management practices may not effectively cater for the full range of specific movement syndromes, resulting in a bias in favourable conditions and proliferation of a certain movement syndrome, thereby possibly leaving the population less resilient. Alternatively, the presence of multiple movement syndromes may also mean a broader, more flexible range of management strategies may be appropriate for at least a portion of the population. While the functional significance of these movement behaviours is not yet fully understood, their persistence within the population suggests that each of these strategies is viable and may play an important role for the overall population. Catering for these movement strategies by updating management practices, such as hydrologic connectivity through environmental flow releases, could create better outcomes.

Movement ecology studies in freshwater fish generally aim to quantify the flow-related requirements necessary to facilitate essential life history events, such as spawning and migration [34, 77]. While the findings of this study do not directly define flow requirements for specific life-history events, they suggest that diversity in movement behaviours within a population may provide some resilience in river catchments with high hydrological variability and flow alteration. Nevertheless, management actions could continue to maintain opportunities for critical life-history processes, including reproductive and dispersal movements, to ensure the persistence of the full range of movement syndromes within the population. Preserving this behavioural diversity is likely important for maintaining long-term population resilience under ongoing flow alteration and climatic variability.

## Supplementary Information

Below is the link to the electronic supplementary material.

Supplementary Material 1

Supplementary Material 2

## Data Availability

All data associated with this study will be made available upon request to the corresponding author.
