# Supplementary material for: Age, morphology, and environmental variation shape movement behaviour syndromes in a riverine fish – golden perch (Macquaria ambigua)
Source: Mov Ecol. 2026 Jun 12;14:54. doi: 10.1186/s40462-026-00672-8 (PMC13417841; doi:10.1186/s40462-026-00672-8)
Supplement: Supplementary file 1 — Supplementary Material 1 [file 40462_2026_672_MOESM1_ESM.docx]

**Supplementary Information for Movement characteristics of Golden perch (Macquaria ambigua) and the influence of age, morphometric traits, and environmental variation in the Condamine–Balonne River.**

Supplementary Table 1. Biological metrics and capture details of golden perch from the Condamine-Balonne River. (See “Supplementary Table 1.csv”)

Supplementary Table 2. The relevant Department of Local Government, Water, and Volunteers (DLGWV) and WaterNSW water monitoring stations used in this study

| Source | Gauge number | Name | Latitude | Longitude |
| --- | --- | --- | --- | --- |
| RDMW | 422220A | Balonne River at Surat | 27°09'00.2"S | 149°03'49.7"E |
|  | 422201F | Balonne River at St. George | 28°03'40.9"S | 148°33'48.9"E |
|  | 422213A | Balonne River at Weribone | 27°19'06.7"S | 148°49'32.6"E |
|  |  |  |  |  |
|  | 422308C | Condamine River at Chinchilla | 26°48'00.4"S | 150°34'29.8"E |
|  | 422325A | Condamine River at Cotswold | 27°05'07.9"S | 149°47'11.6"E |
|  | 422333A | Condamine River at Loudouns Bridge | 27°13'30.4"S | 151°11'08.6"E |
|  | 422336A | Condamine River at Brigalow | 26°54'15.4"S | 150°46'58.8"E |
|  | 422344A | Condamine River at Bedarra | 26°50'30.0"S | 150°20'39.4"E |
| WaterNSW | 422015 | Culgoa River at Brenda | 29°1'46.6"S | 147°18'48.5"E |
|  | 422016 | Narran River at Wilby Wilby | 29°26’44.52"S | 147°31’40.8"E |

**
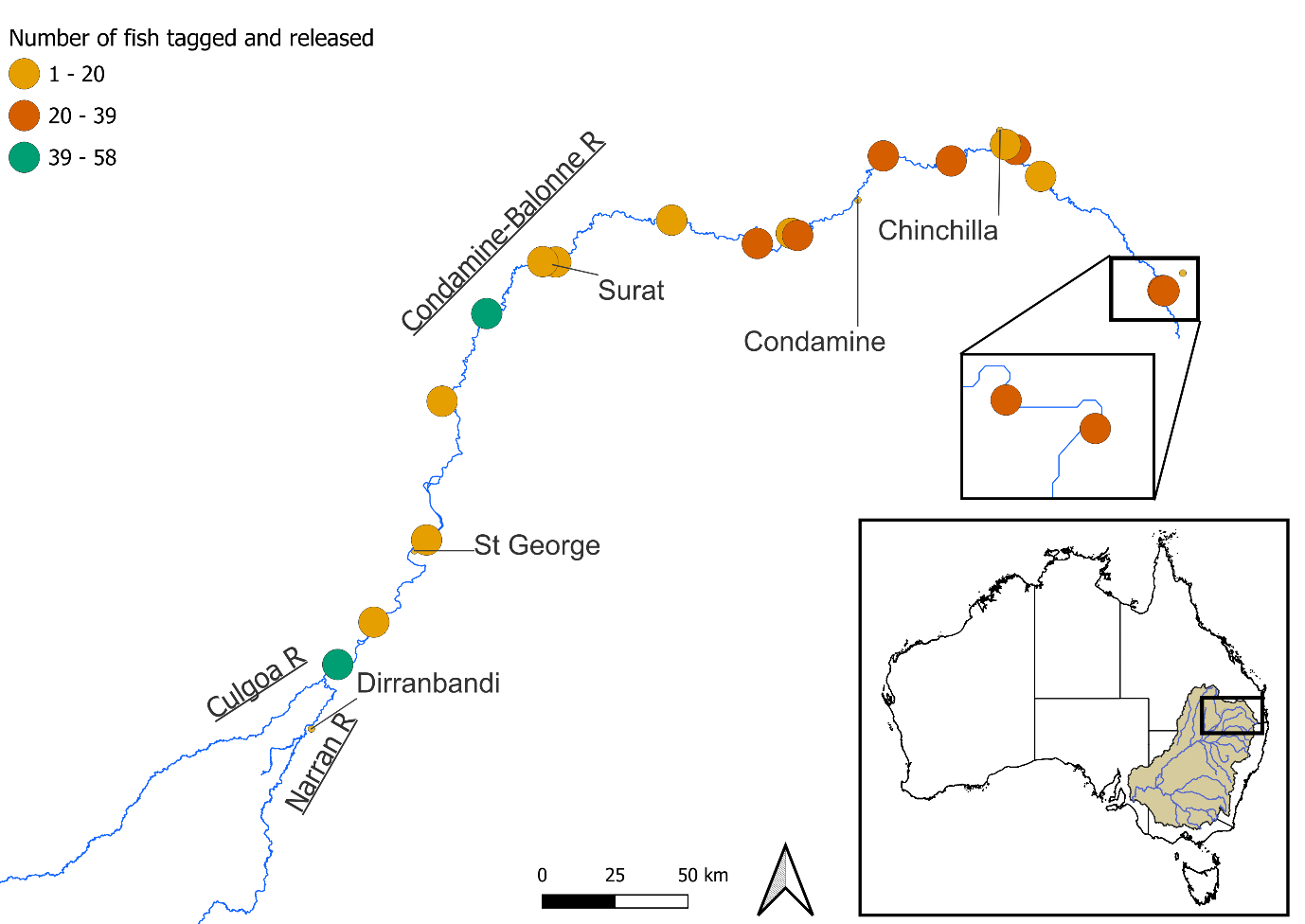
**

Supplementary Figure 1. The number of fish tagged and released at each of 18 sites within the acoustic telemetry receiver array in the Condamine-Balonne River, northern Murray-Darling Basin.


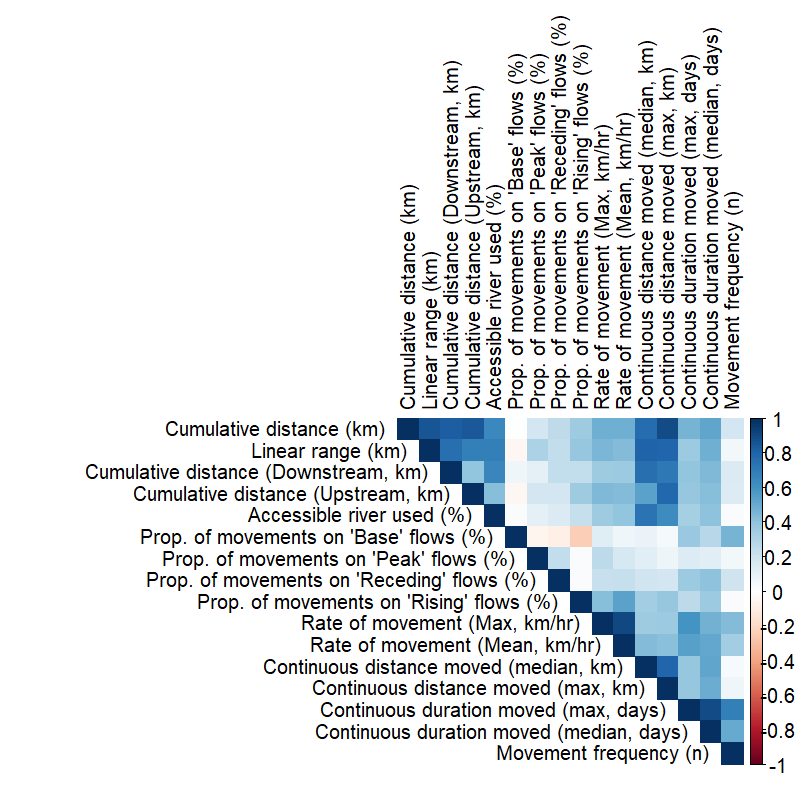


Supplementary Figure 2. Correlation matrix of 16 movement metrics used to characterise the movement behaviour of golden perch in the Condamine-Balonne River. Each cell represents the Pearson correlation coefficient between pairs of variables, with values ranging from -1 (strong negative correlation) to +1 (strong positive correlation). Colour intensity and direction indicate the strength and direction of the relationship.

**Otolith aging using age-length key**

Fish age was attributed to fish based on length at the time of tagging using an age-length key. This was developed as described in Isermann & Knight (2005) using the *FSA* package in R to predict the age of fish based on their lengths (Ogle, 2017) (Supplementary Figure 3). A total of 514 otolith-aged golden perch sampled from 11 sites in the Queensland Murray-Darling basin, including nine sites in the Condamine-Balonne system (Marshall et al., 2025) were used for this purpose, ranging from 46 – 438 mm (standard length) and 0 – 15 years of age. The age-length key was used to estimate the age of the fish tagged in this study by assigning a probability to each age-length interval (20 mm). For example, a fish between 60 – 80 mm is assigned a 58% chance of being 1+ years of age and a 42% chance of being 0+ years of age. The ALK will then randomly assign an age of 0+ to 42% of the 60 - 80 mm fish and 1+ to the remaining 58% of fish in that length interval. The range of length intervals was set at 40 to 420 mm, as there were no known-age fish outside of this range. See supplementary Table 1 for the estimated ages of all golden perch used in this study.


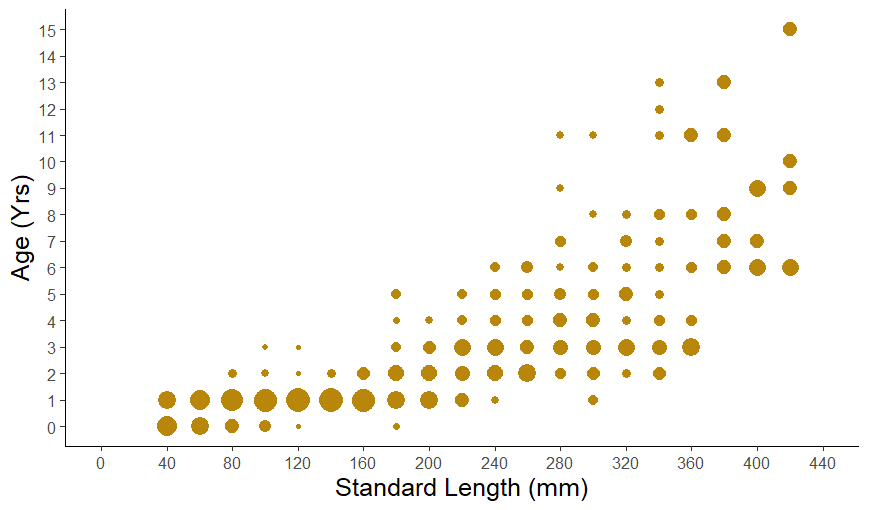


Supplementary Figure 3. Modelled age-length key for golden perch in the Condamine-Balonne River developed from otolith-aged golden perch sampled in the Condamine-Balonne River (*n* = 514). Bubble size indicates the proportion of golden perch at that age (years) at each 20 mm length interval.


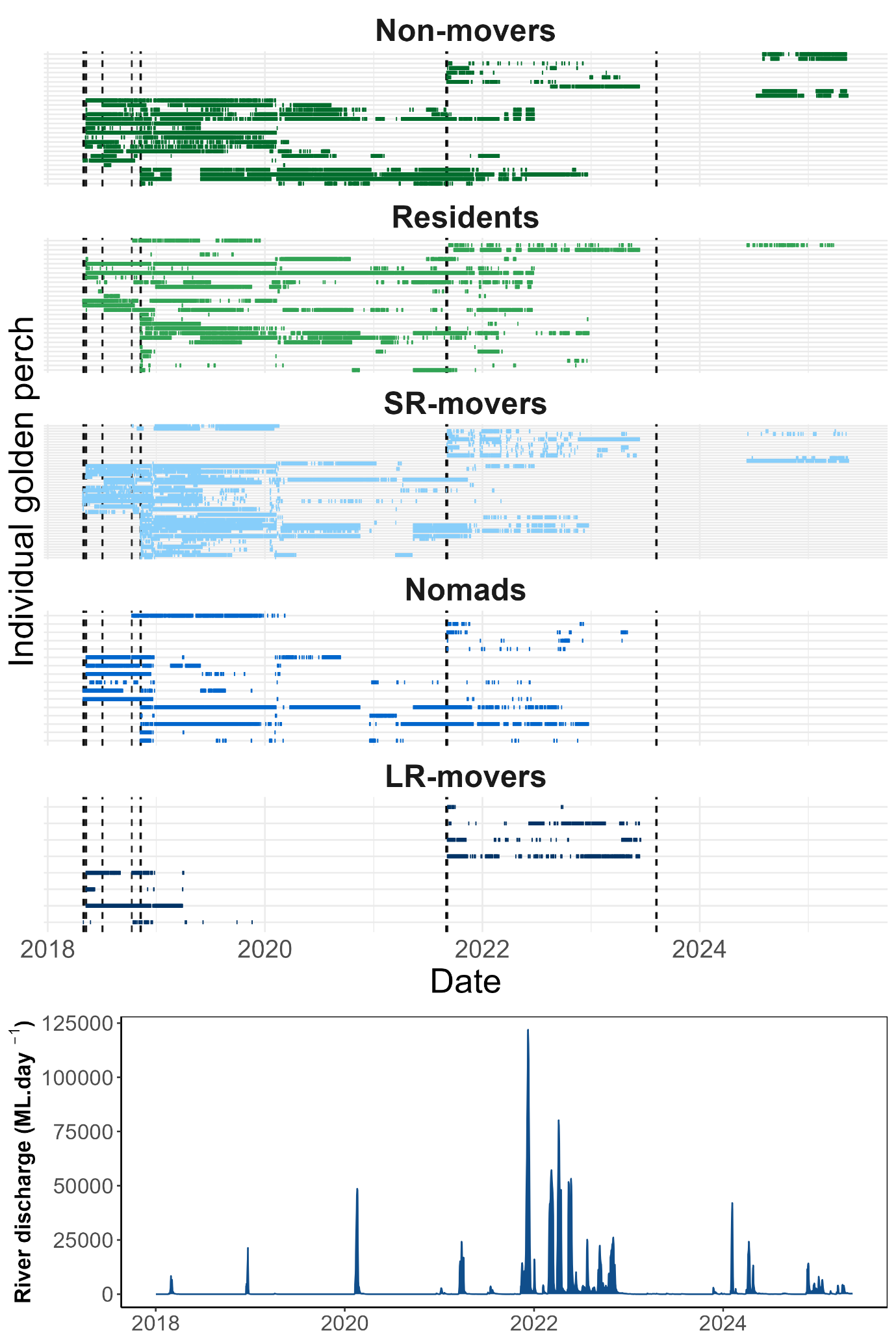


Supplementary Figure 4. Detections of tagged golden perch in the Condamine-Balonne River through time, separated by five identified movement syndromes (top). Vertical dotted lines denote tag and release events. Daily flow discharge (ML/day) throughout the study period (bottom).
